# Revisiting the Cutaneous Impact of Oral Hormone Replacement Therapy

**DOI:** 10.1155/2013/971760

**Published:** 2013-12-21

**Authors:** Gérald E. Piérard, Philippe Humbert, Enzo Berardesca, Ulysse Gaspard, Trinh Hermanns-Lê, Claudine Piérard-Franchimont

**Affiliations:** ^1^Laboratory of Skin Bioengineering and Imaging, Department of Clinical Sciences, B23, University of Liège, 4000 Liège, Belgium; ^2^Department of Dermatology, University Hospital St Jacques, 25030 Besançon, France; ^3^San Gallicano Dermatological Institute, Department of Dermatology, 00144 Roma, Italy; ^4^Department of Gynecology and Obstetrics, University Hospital of Liège, 4000 Liège, Belgium; ^5^Department of Dermatopathology, Unilab Lg, University Hospital of Liège, 4000 Liège, Belgium; ^6^Department of Dermatology, Regional Hospital of Huy, 4500 Huy, Belgium

## Abstract

Menopause is a key point moment in the specific aging process of women. It represents a universal evolution in life. Its initiation is defined by a 12-month amenorrhea following the ultimate menstrual period. It encompasses a series of different biologic and physiologic characteristics. This period of life appears to spot a decline in a series of skin functional performances initiating tissue atrophy, withering, and slackness. Any part of the skin is possibly altered, including the epidermis, dermis, hypodermis, and hair follicles. Hormone replacement therapy (oral and nonoral) and transdermal estrogen therapy represent possible specific managements for women engaged in the climacteric phase. All the current reports indicate that chronologic aging, climacteric estrogen deficiency, and adequate hormone therapy exert profound effects on various parts of the skin.

## 1. Introduction

Aging is a heterogeneous multifaceted process exhibiting different aspects among animal species and humans. Distinct aging classifications were thus offered to biologists. One important distinction recognized the dual presentation and evolution of germ lineages and somatic cells. Human senescence is characterized by distinct processes affecting the somatic and germ lineages. Considering species with different cell lineages and distinct life histories among tissues, the aging process needs to be adequately qualified [1].

A distinction is commonly drawn between aging affecting men and women. It stems from major biologic gender differences. A variety of intra- and extracellular molecular compounds are commonly but distinctly involved during gender-related human aging. One of the major issues concerns estrogen depletion at menopause. Such a feature likely exerts a prominent influence on women aging in a variety of body systems including, the cardiovascular system, brain, bones, joints, and skin [2, 3]. Some physical manifestations progressively take root at menopause. They commonly include vasomotor instability, urogenital atrophy, palpitations, and headaches, as well as bone and joint tenderness, asthenia, disturbed sleep, tiredness, breast tenderness, and skin outlook decrement [4].

Natural menopause is clearly a “one-off” process initiated during the first 12-month amenorrhea following the ultimate menstrual period. The average menopause age is about 49–51 years, corresponding to the decrement in and loss of ovarian cyclic activity. Of note, a number of conditions potentially trigger a premature occurrence of menopause. The transition phase from regular ovarian cycles to menopause is not a swift biologic event. Rather, a series of progressive hormonal and clinical alterations take place during the progressive decline in ovarian activity [5]. The elapse period between the end of the reproductive life and the early stages of postmenopausal phase is referred to as perimenopause or climacteric. It refers to the surrounding years both before and after menopause itself when the process of ovarian decline supervenes associated with endocrine and concurrent physiologic changes. Postmenopause corresponds to the initial year of menopausal amenorrhea and the time thereafter.

The main ailments and medical conditions attributed to menopause correspond to an increased frequency and severity in cardiovascular disease and osteoporosis. Of note, these conditions represent leading causes of mortality in women all over the world. Inevitably, at menopause human skin behaves similarly to any other sex steroid-sensitive tissue. It is commonly subjected to regressive alterations during women climacteric aging (WCA). Of note, WCA appearance is largely perceived through the skin presentation which supposedly reflects at least in part the general health.

## 2. Skin Aging Diversity

In any human organ system, most of the diverse aging processes commonly follow a seemingly linear decrease in both the expected upmost functional activity and the potential reserve capacity. However, WCA is not linked to a similar timetable because it is initiated as a turning point at the entrant process in women's aging. It is further noteworthy that skin WCA is associated with a variety of other age-related physiologic feeling of weakness issued from a series of other intrinsic and environmental threats [6].

The restricted dual presentation of chronologic and actinic skin aging was challenged because of its oversimplification about the global skin evolution in life. A broader list of seven distinct modalities affecting skin aging was offered [7]. The major aging promoters included (a) the elapsed time of life, (b) the endocrine and (c) overall metabolic status, (d) the individual lifestyle, and (e) the cumulative exposures to electromagnetic radiations, as well as diverse repeat mechanical promptings from (f) muscle contractions and (g) extrinsic forces including earth gravity (Table 1). Accordingly, WCA of the skin is part of the global endocrine aging process. Recent years have witnessed progresses in understanding the hormonal involvement in the overall aging process [8–10]. A gender perspective is thus clearly required for a full understanding of skin aging [9, 10].

In such a context, the optimal physical appearance, as well as the structural integrity and functional capacity of the skin tissues require the adequate balance between a number of endocrine influences [6, 8]. Any disturbed activity in the naturally controlled endocrine system potentially abates some skin performances [8]. Estrogens and other sex steroids particularly influence both the skin tissue compositions and structures [3, 9]. In addition, any decreased estrogen production during menopause exacerbates a series of other deleterious age effects in women.

## 3. Skin Climacteric Aging

Skin represents a major interface between internal organs and the environment. It is a complex intricate structure exhibiting a set of mechanisms connected to the rest of the body. In particular, it reflects a number of internal characteristics of the body. It is obvious that a series of genetic and hormonal influences affect the skin structures and functions causing gender variations to change with age.

In many Western social groups, a rapid increasing trend is recognized in the aging segment of the population, particularly among women. In recent decades, some research priorities targeted both the physiology and management of WCA [11]. The regular progress in women life expectancy led to the increased growth of this population living long after menopause. Nowadays, women expect to live about one third of their lives in a potential estrogen deficient state. Overall, extended longevity coupled with the fact that women statistically outlive their partners have generated diverse and special needs in women. The potential WCA impact is present at the origin of a series of physiologic changes and age-related diseases.

Before menopause, the regular ovulatory cycle is controlled by complex interactions between diverse endocrine signals from the hypothalamus, pituitary gland, and ovaries. It is further modulated by the brain cortex, as well as by hormones issued from the adrenal and thyroid glands, and by some peripheral hormone productions as well. Each ovulatory cycle corresponds to the simultaneous activation of a few ovarian follicles. Among them, one single follicle usually becomes dominant and leads to ovulation. In the women reproductive period, about 200,000 ovarian follicles give rise to 500 or so mature ovocytes. Hence, ovarian follicular atresia corresponds to a physiologic dominant process and represents a key process leading to menopause [2]. During the regular ovarian cycle, estradiol represents the physiologic dominant estrogen, reaching a peak level at ovulation. A mid-cycle uprise of blood levels in the follicular stimulating hormone (FSH) and luteinizing hormone (LH) occurs typically.

There is evidence that estrogens and other sex steroids impact many organ systems [2, 5, 9, 10]. In particular, they influence both the skin composition and biology [5, 10]. The gender influence and the regional skin depositions of estrogens vary considerably. In particular, estrogen receptors (ER) were identified at a higher density in women than in men. They appeared unevenly scattered among different body locations, with larger densities on genital target organs, the face, and legs. In these locations, they were particularly identified in the epidermis, sebaceous hair follicles, and sweat ducts [2, 12, 13]. They probably influence the overall corneocyte sizes [14]. By contrast, ER appeared less abundant or almost absent in the dermis and sweat glands. A high ER/androgen receptor ratio is found in the vagina, while the reverse ratio exists in the vulva. Both ER and progesterone receptors decline in the skin from the climacteric onwards [2, 12, 13].

The functional ovarian activity fails during the early stages of WCA. During the menopause transition, antimullerian hormone, and inhibin B, markers of follicular reserve and function vanish dramatically and anticipate the progressive ovarian follicular demise [14]. At menopause, a significant change takes place in the sources and amounts of estrogens. Indeed, during the reproductive period, the main part of estrogen production originates from the active ovarian follicles. After menopause, most circulating estrogens are derived from the peripheral conversion of androstenedione to estrone. These changes occur gradually with age. Aromatase activity remains present in fibroblasts, adipocytes, and sebocytes of postmenopausal women.

Of note, a dramatic increase in FSH takes place at menopause. A slow decline follows over the next decades. Simultaneously, a somewhat modest increase occurs in LH levels [15]. Contrasting with the decline in estrogens and progestins, androgen production remains almost unaffected during menopause transition. Indeed, circulating amounts in testosterone, androstenedione, dehydro-epiandrosterone (DHEA), and DHEA-sulfate (DHEAS) are not markedly altered during premenopause. However, during postmenopause, they decline slowly to about 50% of their premenopause levels. The postmenopausal ratio androgens/estrogen increased compared to premenopause [16].

## 4. HRT and Skin Climacteric Aging

For about 70 years, the rationale for hormone replacement therapy (HRT) in WCA appeared straightforward for many physicians [17–21]. The positive effect of HRT was first demonstrated in a large observational cohort study started in the midseventieth. Therefore, health providers encouraged women to use HRT as prevention strategy for cardiovascular disease. Furthermore, the HRT impact on skin thickness and dermal density was demonstrated early when estrogens were initially administered to postmenopausal women. Such replenishment therapy was therefore considered as an attempt at alleviating in part skin atrophy and xerosis in postmenopausal women [2, 5, 6]. However, it remains that menopause and its specific HRT have not yet resolved a number of other WCA challenges. Some reluctance emerged in prescribing HRT. The concern was recently documented, particularly in the United States, pointing to adverse events, particularly venous thromboembolism and absence of overall protective benefit against coronary heart disease associated with high levels in administered estrogens. Other reports from Europe were less disturbing. Indeed, women profile and hormones used in Europe were different from the US. However, no randomized, controlled trial using European hormone therapy has been performed and no definite answer concerning the risk and benefit ratio of HRT on cardiovascular risk exists. Anyway, some discrepancies persist in the current literature. Globally, nowadays HRT appears to improve a series of climacteric changes in diverse organs including the skin [22–24]. Recent studies confirmed that both estrogens and estroprogestins in part suppressed signs of the climacteric syndrome including genital atrophy and the risk of osteoporotic fractures [18, 19].

The perimenopause estrogen depletion likely contributes to and exacerbates the negative effects of aging. It seems obvious that cutaneous changes developed during the initial postmenopausal decade are both age- and hormone-related [16, 25, 26]. Indeed, postmenopausal women commonly complain of xerosis, easy skin withering, bruising, and wrinkling [8]. In many instances, the climacteric impact on skin, and its HRT correction proved to be particularly difficult to objectivate and be quantified by clinical examination alone [24, 27]. It remains that some relevant aspects were conveniently rated on semiquantitative scales. Visual and tactile perceptions of the skin condition still represent valuable tools in clinical practice. However, they suffer from limited sensitivity, specificity, and reproducibility in comparative assessments performed over a long-term period of time. In addition, the clinical appearance is sometimes misleading compared to the actual biologic effects of WCA and HRT. By contrast, noninvasive objective methods of biometrology are probably better suited for improving the reliability and precision of in vivo skin assessments [27]. During the last few decades, quantitative measurement methods used in dermatological research improved substantially, providing means of relevant evaluation of skin functions and characteristics. By this way, some WCA skin alterations are readily identified during incipient menopause. It remains difficult to distinguish the consequences of menopause from other age-related changes due to a decline in growth hormone [8]. Indeed, depletions in both estrogens and growth hormone are combined in that period of women life.

For years, HRT effects on the skin have deservedly attracted interest [2, 24, 25, 28], but evidence-based issues remained unsettled or controversial [25, 29, 30]. It seems that short term HRT treatments fail to bring marked improvements on the skin structure [29, 30]. The maximum prevention at skin aging appears to occur following HRT administration during the perimenopause [2, 22, 31]. The value of phytoestrogens and the possible implication of some hormone disruptors from the environment are not established so far in skin WCA.

## 5. HRT and Skin Epithelia

The perception of WCA is commonly based on the appearance of the skin surface. Globally, it depends on the process of stress-induced premature senescence (SIPS) results from a series of distinct sublethal threats including the effects of reactive oxygen species and those of a variety of other chemical insults [32]. Changes include among others some alterations in the morphologic presentation, as well as senescence-associated disturbed enzyme activities, cell cycle dysregulations, disturbed gene expressions, and telomere shortening. Cells engaged in replicative senescence share common features with those involved in SIPS [32]. In replicative senescence, telomere shortening is associated with accumulations of DNA single-strand breaks induced by oxidative stress. Thus, SIPS probably contributes to the in vivo accumulation of senescent-like epidermal cells. Furthermore, DNA damages play a key role in both the regular chronologic skin aging and photoaging [33]. Estrogen depletion possibly promotes and activates SIPS [34].

In any subject, the skin surface is traversed by furrows that intersect in complex ways, creating geometric patterns. The skin surface markings vary with age. In young women, the patterns are quite orderly. In WCA, the furrows become shallower and overall plateaus appear larger with some loss in regularity, but the overall geometry is retained.

Severe forms of WCA are associated with a dramatic flattening of the epidermis associated with retraction of the rete ridges. The keratinocyte renewal is decreased. The keratinocyte maturation is altered with formation of xerosis represented by a more compact aspect of corneocyte clumps. Xerosis is a stratum corneum disturbance interpreted as “dry skin” by laypeople. This condition indeed results from altered desquamation often followed by a decreased hydration of the uppermost corneocytes and a weakening of the skin barrier function. Both the water-holding capacity and barrier function of the xerotic stratum corneum were reported to be restored at least in part following HRT [25, 35, 36]. All these changes are associated with alteration in the epidermal cell renewal.

Lip mucosa structure and functions are quite distinct from those of the surrounding skin. Lip mucosa is subjected to repeat mechanical promptings and to other physical and chemical stresses. Its age-related structural changes were reported to be associated with alterations in its potential extension and contraction [37, 38]. In addition, the hydration of the lip surface was markedly different between both lips. It was claimed that any hormonal effect was unlikely in the age-related changes in lip surface hydration and lip mechanical properties [38]. Such contention remains yet unsettled.

## 6. HRT and the Dermal Extracellular Matrix

It is acknowledged that the dermis thins with aging including WCA. Accordingly, the individual dermal components have received much attention in an effort to identify markers of dermal aging in women. In young individuals, the dermis is a tough connective tissue composed of networks of fairly stable fibers, predominately and distinctly composed of collagen and elastin. Fibrous collagen represents about 80% of the dry weight of the dermis in young adults. Its fibers are characterized by a high tensile strength and they prevent the skin from being severely torn by usual stretching. Collagen density represents the packing and compactness of fibrils inside bundles. Such a structure commonly becomes distorted following repeat and life-long mechanical constraint. Elastic fibers make up near 5% of the dermis on sun protracted areas. They act to recoil the skin to its rest position after deformation. Dermal cells corresponding to fibroblasts and dermal dendrocytes synthesize and control most components of the dermal extracellular matrix. Their numerical density globally declines with aging when they commonly evoke thin fibrocytes with a shrunken cytoplasmic volume suggesting some abated metabolic activity.

The dermis commonly shrinks with WCA. The reduction in dermal collagen packing and content was reported to occur rapidly after menopause and to gradually progress thereafter [2]. It was assumed that about 30% of the dermal collagen vanished in the initial 5 postmenopausal years. This prominent loss was followed by a slower average 2% yearly decline over the following two decades [2]. Collagen networks on sun-protected skin areas are thinner and less compact in aged people.

The interstitial extracellular matrix present between collagen bundles contains a mixture of hyaluronic acid, versican, and other glycosaminoglycans [39]. Dermal water is bound to these hydrophilic compounds. Such molecular association protects in part the skin against excessive tissue compression whilst maintaining its suppleness and preventing atrophic withering and slackness. Estrogens possibly boost the dermal hygroscopic properties through enhanced synthesis of dermal hyaluronic acid [2]. A possible role for intracellular versican is further possible although not firmly established [39].

The global integration of the morphologic, molecular, and biophysical information gained on the dermal WCA remains currently somewhat confusing due to the complexity of quandaries. The same problems exist in the understanding of the clinical dermal changes which is at best rudimentary.

One major WCA changes in the dermis involves the organization of the individual collagen bundles. They do not anymore appear in discrete rope-like bundles of tightly packed fibres. Rather they progressively appear as aggregates of loosely women fibers. The overall rearrangement of the dermal collagen network is responsible for the changes in the biomechanical properties of the dermis in menopausal women.

Some facets in HRT contribute to changes in the dermal physical properties of menopausal women. Indeed, HRT controls in part the dermal thickness and laxity, and the collagen content and density, as well as the tissue mechanical reactivity to stress [2, 23]. From an engineering point of view, human skin represents a complex load transmitting structure. It is subjected to self-originated and environmentally imposed responses. Modalities of HRT administration differed among trials over time. Diverse estrogens were administered singly or in combination with cyclic administration of progesterone-derived compounds. The information about skin effects was commonly discussed collectively without distinguishing these modalities.

At present, no consensus has been reached about the real value of HRT on dermal climacteric aging. Combined molecular biology and morphology have deferred comprehension of structural changes occurring with aging in the collagen network and density. Globally, the dermal thickness, its collagen density, and content were reported to be possibly maintained in HRT receivers compared to age-matched untreated women [2, 22]. However, a few other authors denied any significant HRT effect on the dermal collagen [29, 30]. Still other researchers contended that various levels of skin response to HRT were distinguished. These conditions encompassed good and poor responders [31–33], the latter category possibly corresponding to smokers or to women recently entered in the climacteric period without presenting a loss in estrogen-replaceable collagen [2].

In menopausal women presenting a low collagen density in the dermis, estrogen replenishment is expected to initially correct and later exert a prophylactic action. By contrast, women with mild reduction in the skin collagen amount during the initial menopause phase, estrogens apparently exhibit a prophylactic effect only [2, 40]. Therefore, any depletion in skin collagen content is expected to be in part normalized although not overstimulated. It was claimed that the skin collagen replenishment exhibits regional variability with an increased effect on the abdomen [2].

Climacteric appears to be responsible for atrophic withering, wrinkling, and slackness, particularly on the forearms and face. Atrophic withering, fine wrinkling and progressive deepening of facial creases ensue. The quantitative decrease in collagen density in the dermis probably participates to the progressive climacteric skin slackness. Such alterations were reported to be partially reversed in HRT-treated postmenopausal women [40, 41]. Increased skin distensibility and impaired elasticity commonly develop in normal weight untreated perimenopausal women [27, 42, 43], and this feature is mitigated by HRT. Such correction helps preventing skin slackness [41]. Deepening of wrinkles is commonly associated with these functional changes. In some instances, HRT exerts a beneficial effect on the facial skin aspect by reducing the age-related rheological changes without, however, limiting the number and depth of wrinkles [26, 41].

## 7. HRT and the Dermal Microvasculature

The dermal arterioles, capillaries, and venules differ from the microvasculature of other organs due to the distinct thickness of the vascular vessels. Estrogens appear to control the skin vascularization in women [44–46]. Menopausal skin flushes reflect prominent vasodilation particularly on the face, neck, chest, palms, and soles. They correspond to an active process depending in part on the net effect of the dermal fibrous rearrangements. The flush prevalence during early climacteric is probably more reported to the estrogen depletion, with a resulting loss of control in the peripheral microvasculature tonus. The flush phenomenon is expected to fade at least in part following HRT. The maximum inducible vasodilation was reported to be reduced in climacteric women receiving HRT compared with untreated postmenopausal women [44]. The beneficial effect of HRT on the skin blood flow has, however, been challenged. HRT users have possibly fewer chronic leg ulcers and pressure-induced ulcers [47]. In the elderly, estrogen might increase the wound healing rate. This finding warrants confirmation before recommending HRT to improve wound healing.

## 8. HRT for Skin and Bones

Osteoporosis is a major feature of WCA. It is commonly associated with clinical manifestations on the skin presenting as atrophic withering, wrinkling, sagging, and laxity. A correlation was reported between skin biomechanical properties and bone densities [42, 48]. The combination of decreased skin thickness, altered dermal biomechanical functions, and loss of bone mineral density probably presents the greater sensitivity and specificity in identifying women postmenopausal vulnerability to osteoporotic fractures.

The potential relationship between osteoporosis and dermatoporosis is probably important to be noted. The similarity of HRT effects on both conditions would be important to be adequately explored in the future.

## 9. Conclusion

The few past decades have witnessed a markedly increased interest in skin aging on the part of health care providers. The particular interest in skin WCA paralleled the constant rise in the number and proportion of women living beyond menopause. A number of societal issues result from this demographic shift.

Global aging results from the cumulative and synergistic effects originated by each of the seven specific aforementioned causes. The older concept of skin aging immutability is partially challenged by this way. Increased awareness in the distinct aging inducers in the skin including WCA potentially leads to more business-like skin care managements and promotes innovative developments of specific preventive measures.

Clearly, the adequate nature and levels of sex steroids influence both the skin structural integrity and its optimal functional capacity. Any reduction and failure in this hormone activity exert a key role in WCA as suggested by the progressive decline in skin appearance from perimenopause onwards. Indeed, most women clearly associate climacteric with a decline in skin presentation and a global negative feeling in life experience. In particular, a series of skin changes and failures come to develop at menopause and get worse afterwards. Yet, some of these aspects remained neglected for a long time by the biomedical research. However, investigations about the climacteric and postmenopausal aging of the skin were initiated in the recent past decades. WCA is being recognized as a significant and treatable health problem.

Clinical strategies in HRT have advanced in sophistication over time. It remains that people either conducive or opponents to HRT contribute to render it a complex issue. It remains that HRT administration appears quite effective on skin, provided adequate patient selection (body weight, cancer risk, etc), respect of contraindications, and appropriate hormones including their nature, dosages, regimens, and routes of administration. The average HRT duration is roughly 3 years. HRT commonly increases the well-being and corrects a series of somatic features in menopausal women. The effectiveness and increased safety of HRT are expected in the future. All the foregoing aspects indicate that the combination of chronologic aging, the climacteric estrogen deficiency, and HRT exert profound and distinct effects on various aspects of skin physiology. In many instances the deleterious effects of low estrogen amounts on the skin show concurrent manifestations in diverse internal tissues and organs.

HRT trials are frequently associated with disappointing compliance. Hesitation to initiate HRT and discontinuation are commonly experienced. In compliant women, HRT commonly appears to protect in part the skin from some of the negative WCA changes.

Integration of updated concepts into patient management should prove to be critical to optimizing prevention, diagnosis, and management of WCA and its related dermatoses. HRT acts in direct or probably indirect ways on several distinct skin structures. The effects are mediated by direct hormonal effects on cells harbouring the adequate specific receptors. Some hormone-stimulated target cells produce in a second step paracrine signals to other cells which thus become indirectly influenced by HRT. With HRT signalling pathways, the epidermis is boosted, and the dermal water content is enhanced. As a result, skin represents a target organ where HRT benefits are possibly noticeable by the women and their relatives.

Adverse reactions to HRT are probably the most frequently observed on the skin and breasts including venous thromboembolism. Knowledge of the complications helps to prepare the potential patient, to reassure wavering women, and to avoid annoying skin reactions. Thus the HRT compliance is likely improved, and the number of women enjoying some beneficial effects of HRT is potentially increased.

## Figures and Tables

**Table 1:** Cutaneous aging modalities (from [7]).

Aging parameters	Determinant factor
Chronology	Elapse time
Genetic	Genetic (premature aging syndromes, melanotype-phototype-related)
Actinic	Ultraviolet and infrared irradiations
Behaviour	Diet, tobacco, alcoholic abuse, drug addiction, and facial expressions
Endocrinology	Pregnancy, physiologic, and hormonal influences (ovaries, testes, thyroid, adrenals, growth hormone, etc.)
Catabolism	Chronic intercurrent debilitating disease (infections, cancers)
Gravitation	Earth gravity
